# Licensed and unlicensed cannabis outlets in Los Angeles County: the potential implications of location for social equity

**DOI:** 10.1186/s42238-022-00120-5

**Published:** 2022-04-11

**Authors:** Caislin L. Firth, Kristin M. Warren, Lilian Perez, Beau Kilmer, Regina A. Shih, Joan S. Tucker, Elizabeth J. D’Amico, Eric R. Pedersen

**Affiliations:** 1 Psychiatry and Behavioral Sciences Department, University of Washington School of Medicine, 1959 NE Pacific St, Seattle, WA 98195 USA; 2 RAND Corporation, Santa Monica, CA USA; 3 RAND Corporation, Arlington, VA USA; 4 Department of Psychiatry and Behavioral Sciences, Keck School of Medicine, University of Southern California, Los Angeles, CA USA

**Keywords:** Cannabis, Social equity, Cannabis outlets, Dispensaries

## Abstract

**Background:**

Cannabis social equity programs intend to redress inequities experienced by low income and Black, Indigenous, and People of Color (BIPOC) during cannabis prohibition in the United States. In Los Angeles County (LA), the approach is to increase cannabis outlet licensure and employment for low income and BIPOC communities. Monitoring locations of both licensed and unlicensed outlets over time is critical to informing how local social equity programs may affect communities.

**Methods:**

We identified locations of licensed and unlicensed cannabis outlets in LA, from February to April 2019 and again from March to April 2020, and calculated the number and type of outlets by socio-demographic characteristics of census tracts (race/ethnicity, poverty, education, unemployment) using the 2013–2017 American Community Survey 5-year estimates.

**Results:**

Licensed outlets increased in LA from 162 in 2019 to 195 in 2020; unlicensed outlets decreased from 286 to 137 over the same time period. In 2020, more licensed outlets were in tracts with majority white residents and adults with at least a bachelor’s degree; fewer licensed outlets were in tracts with larger Latinx or Black populations, whereas 71% of unlicensed outlets in 2020 were in low-income tracts, and more unlicensed outlets were in predominately Latinx tracts, high poverty and high unemployment tracts, and tracts with more single female-headed households.

**Conclusions:**

Neighborhood-level analyses are an important first step, but more data are needed for comprehensive evaluations of social equity programs—from individual businesses to the communities living nearby—to understand the impacts on low income and BIPOC populations.

## Introduction

More than 40% of the United States (US) population live in states that have passed laws allowing commercial production and retail sales of cannabis for adults (aged 21+ years) for nonmedical purposes. States have passed these laws for multiple reasons, ranging from reducing size of the illicit market to raising tax revenues. More recently, states have added social equity provisions to redress inequities experienced by low-income and Black, Indigenous, and People of Color (BIPOC) who have been disproportionately affected by cannabis prohibition (Resing 2019; Kilmer and Neel 2020), which can have lasting financial and health effects (Health Impact Partners 2016). The effects of these emerging policies on employment, criminal justice, and population health outcomes remain largely unknown. There are multiple approaches to addressing these inequities, through criminal justice reforms, such as, sealing or expunging previous cannabis offenses, and increasing entrepreneurship and employment opportunities to improve economic opportunity and build generational wealth in communities disproportionately affected by cannabis prohibition (Title 2021).

There are important trade-offs to consider when using cannabis policy to address social equity (Kilmer et al. 2021). For example, if licenses for retail stores prioritize people from disproportionately impacted communities, will this lead to a high concentration of licensed stores and cannabis advertising in these neighborhoods, potentially altering norms and increasing health inequities, such as underage cannabis use among BIPOC youth? Research has shown the potential role cannabis advertising can have on underage use. In Oregon, a state with legal cannabis sales for adults (21+ years), three out of four 8th and 11th grade students saw cannabis advertising in the past 30 days, and underage use of cannabis was associated with higher advertisement exposure (Fiala et al. 2020). In California, a study showed that increased cannabis advertisement exposure over 7 years was associated with increased cannabis use and consequences from using (e.g., missing school) among adolescents, even before statewide adult legalization (D’Amico et al. 2018).

The net effect of licensed stores on health remains poorly understood because unlicensed outlets continue to exist in some areas and do not necessarily follow product safety restrictions (e.g., pesticide limits, product dosing) that are required of licensed outlets. Another issue is enforcement against unlicensed outlets, which may be disproportionately operated by BIPOC. Although reducing illegal sales should increase the market share for licensed outlets and increase access to regulated products, a crackdown against unlicensed markets may further exacerbate inequities in the criminal justice system and who benefits from cannabis legalization. For example, racial disparities in arrest rates for cannabis crimes increased after legalization in Washington state, and Black adults were more likely to be arrested for cannabis distribution and sale crimes (Firth et al. 2019). The lack of racial diversity in the cannabis industry likely contributes to these criminal inequities, as 3% of licensed outlets in Washington state are owned by Black people (Leshikar 2021).

To better understand these trade-offs and improve equity within the cannabis industry, it is critical to know locations of both licensed and unlicensed outlets and how these locations have changed over time. Unger and colleagues examined whether licensed and unlicensed outlets were more common in high poverty and minority neighborhoods across California in 2018, the first year of legal cannabis sales (Unger et al. 2020). In that year, unlicensed outlets were more common in Latinx neighborhoods in California, but no patterns were detected for licensed outlets (Unger et al. 2020). The present study builds on that analysis and adds to the sparse literature in this area by bolstering methods for identifying licensed and unlicensed outlets. Specifically, we conducted site visits to outlets, developed a longitudinal outlet database to track changes in outlet locations over time, and summarized the number of licensed and unlicensed cannabis outlets across neighborhood environments varying in socio-economic characteristics. The objective of this study is to document how locations of licensed and unlicensed cannabis outlets vary by neighborhood demographics and to identify who is disproportionately exposed to inform local policies may influence both the distribution and access to cannabis outlets. We specifically focus on neighborhood characteristics of cannabis outlet locations in the most populous county in the United States: Los Angeles (LA) County, California (10 million residents).

### Cannabis legalization in Los Angeles

California first approved medical cannabis in 1996; 20 years later, California voters approved the Adult Use of Marijuana Act, paving the way for the 2017 Medicinal and Adult-Use Cannabis Regulation and Safety Act (MAUCRSA) (California Senate n.d.). MAUCRSA allowed existing medical dispensaries to become licensed outlets and required unlicensed outlets to shut down by January 9, 2019, or face legal ramifications (*Cannabis 101* 2020). Despite regulations, challenges in enforcement persist, and many California outlets continue to operate without licenses (*Cannabis 101* 2020).

In 2018, California’s Cannabis Equity Act was signed into law, which funds local jurisdictions to develop programs that reduce barriers to licensure and increase employment opportunities in the cannabis industry for people disproportionately impacted by prohibition (Holcombe 2018). LA County includes several cities, each of which took different approaches to addressing inequities when implementing legalization. For example, the City of LA secured over $7 million in state funding to prioritize cannabis retail licenses for people formally convicted of a cannabis crime or living in a low socioeconomic area and provide them with financial assistance (e.g., business loans, fee waivers) (Bureau of Cannabis Control California 2020). As of April 2021, the City of LA established the Social Equity Entrepreneur Development (SEED) Grant Program, to provide financial assistance for social equity cannabis license applicants; as of December 2021, SEED awards have not been publicly announced (Department of Cannabis Regulation C of LA 2021). To evaluate City of LA cannabis social equity program, in terms of reducing the number of unlicensed cannabis outlets, potential exposure to unregulated cannabis products, and increasing the number of licensed cannabis retailers owned and operated by low-income and BIPOC, it is necessary to first assess which neighborhoods—and the populations living within those neighborhoods—are disproportionately exposed to unlicensed and licensed retailers. Our analysis examines neighborhood patterns in unlicensed and licensed cannabis outlets in LA County in 2019 and 2020, prior to equity applicants being granted retail licenses, to inform future program evaluations.

### Data and analysis

Data were obtained from a cannabis outlet database to track locations of licensed and unlicensed cannabis outlets in LA County over time (Pedersen et al. 2020). In December 2018 and again in December 2019, we scraped online commercial cannabis registries (e.g., Leafly, Weedmaps), using previously published methods (Pedersen et al. 2020), and collected LA County licensed outlet reports to obtain address information for all outlets in LA County zip codes. We also scraped both websites in June 2019 to assess fluctuations in number and spatial patterning of outlets after the California Bureau of Cannabis Control (BCC) demanded Weedmaps remove listings for unlicensed outlets by January 1, 2020 (Schroyer 2020). Unlicensed outlets were still listed at the time we assembled data sets. We identified 531, 431, and 457 listings in LA County on either Leafly or Weedmaps in December 2018, June 2019, and December 2019, respectively. Our analysis used listings scraped in December 2018 and 2019.

We verified cannabis outlet licenses and conducted site visits in February to April 2019 and again from March to April 2020. We verified licenses for all outlets by reviewing the City of LA Department of Cannabis Regulation–authorized retail business database for outlets within the city (Department of Cannabis Regulation C of LA n.d.) and the License Search Tool on the California BCC website for outlets outside of LA city limits (Bureau of Cannabis Control California n.d.). Outlets that did not have a licensee record in either regulatory database were determined to be unlicensed. We conducted site visits to each outlet address to determine whether the business was currently operating at that location. Site visits were completed by three trained staff members for each wave of data collection; data were recorded on iPads and included a photo of each storefront (Pedersen et al. 2020). After site visits, we determined that 81% (430/531) of outlets in 2019 and 74% (337/457) in 2020 were currently operating; detailed results from 2019 site visits have been previously published (Pedersen et al. 2020).

Operating cannabis outlets were geocoded, resulting in 430 unique addresses in 2019 and 332 in 2020 (five outlets in 2020 were duplicates during the geocoding process; for instance, three outlets were in one strip mall and shared the same street address). We joined census data from the 2013–2017 American Community Survey 5-year estimates with geocoded locations of cannabis outlets; we assessed the number of licensed and unlicensed outlets within each census tract —a spatial unit that represents an average of where 4000 people live, and by socio-demographic characteristics of the tracts. We selected socio-demographic characteristics that represent domains of neighborhood socio-economic status (Escarce et al. 2011) (percentage of households with income below the poverty line, percent of adults aged 25 or older with at least a bachelor’s degree, percent of single female-headed households, percentage unemployed, and median household income) and racial and ethnic composition of the neighborhood. We used univariate linear regression models to assess correlations between the number of licensed and unlicensed outlets in census tracts and each socio-demographic characteristic. We present findings as the number of licensed and unlicensed outlets within each quartile of a socio-demographic measure and compare the number and type of outlets across LA tracts and over time (e.g., we compare the number of licensed outlets in 2020 tracts where the fewest Black people lived [quartile 1] compared to the number of licensed outlets in tracts with the largest Black population [quartile 4]).

## Results

The number of licensed outlets within LA County grew from 162 in 2019 to 195 in 2020 and the number of unlicensed outlets that advertised online decreased from 268 in 2019 to 137 in 2020.

**Fig. 1:**
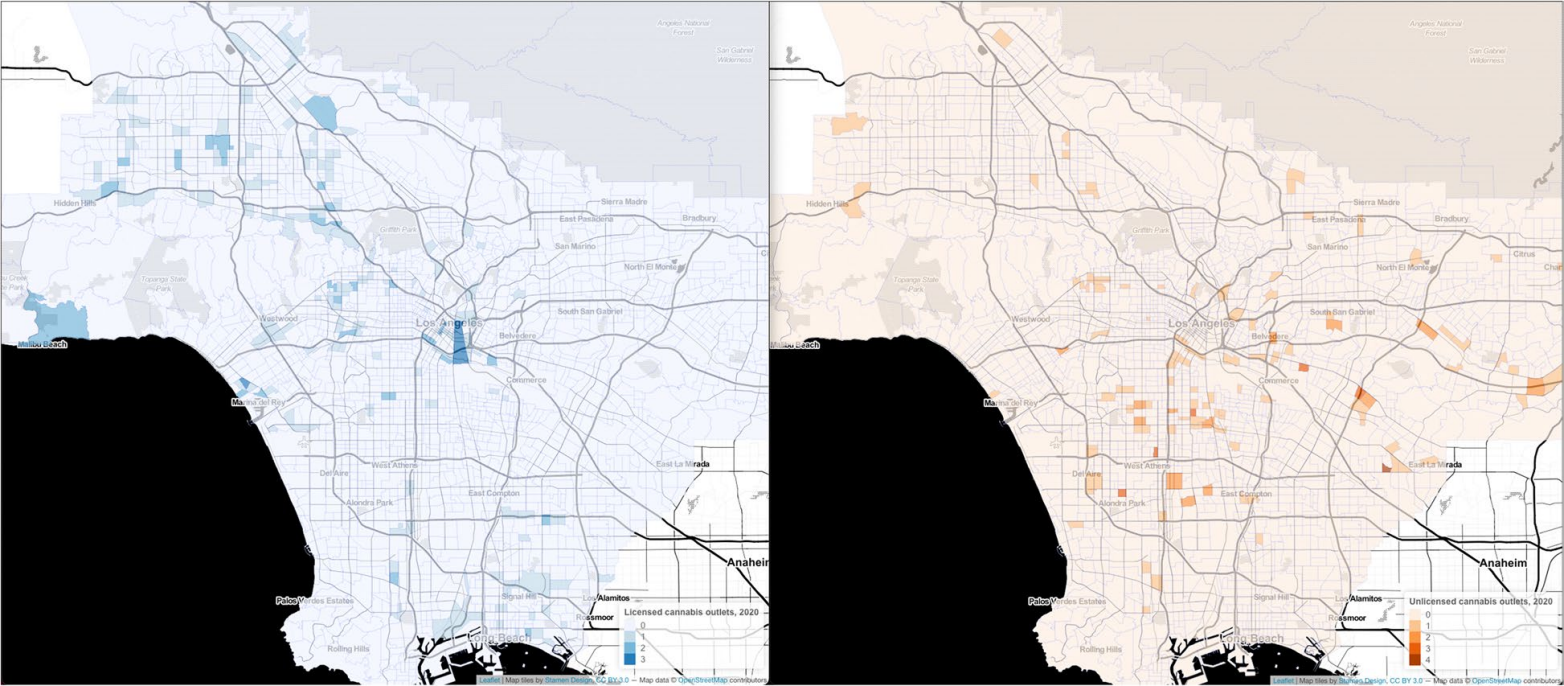
Licensed and unlicensed cannabis outlets in Los Angeles County census tracts, 2020

**Fig. 2:**
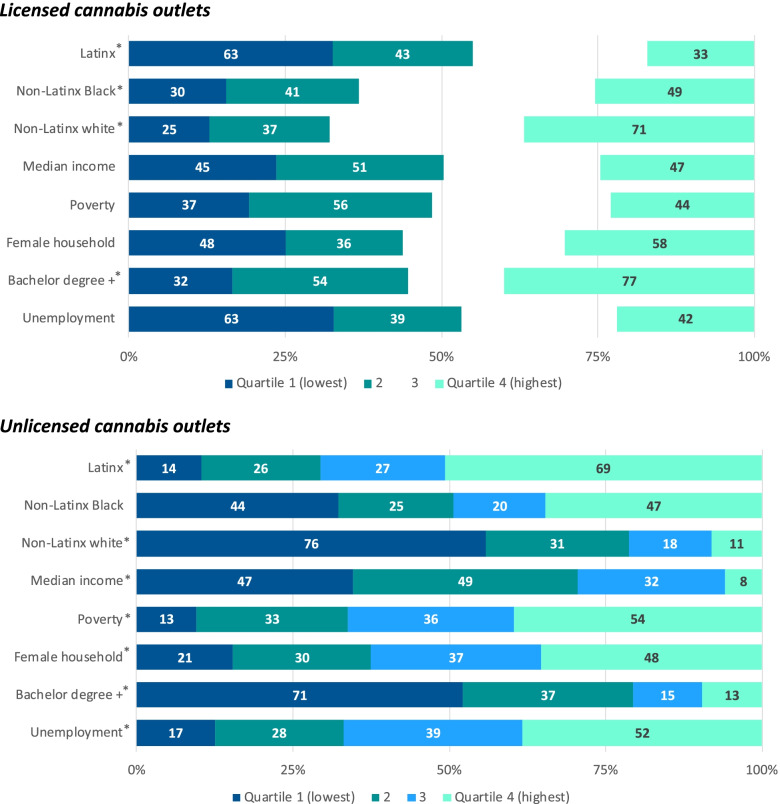
Licensed and unlicensed cannabis outlets in Los Angeles County by census tract socio-demographic characteristics, 2020

Licensed and unlicensed outlets are clustered in different neighborhoods in LA County (Fig. 1), suggesting unequal exposure for different population groups. We found that in 2020 licensed outlets were more common in tracts with majority white residents and with higher proportions of adults with at least a bachelor’s degree and less common in tracts with larger Latinx (includes people who identify as Hispanic, Latino, Spanish origin) or Black populations (Fig. 2).

Despite efforts to shut down unlicensed outlets, their presence remained in LA County. Nearly half of all unlicensed outlets in 2020 were not in the cannabis outlet database in 2019, which suggested that enforcement alone has not prevented new unlicensed outlets from opening. Our data show that 71% of unlicensed outlets in 2020 were in low-income tracts—areas where median household income was below $59,444 (corresponding with number of unlicensed outlets in quartiles 1 and 2 of median household income in Fig. 2). We also found that unlicensed outlets were clustered in predominately Latinx neighborhoods (Fig. 2). Unlicensed outlets were also more prevalent in tracts with the highest rates of poverty, single female-headed households, and unemployment.

## Discussion

In the first 3 years of nonmedical cannabis retail sales in LA County, the number of licensed outlets increased over time, and 137 unlicensed outlets were still operating in 2020. Our data show that licensed outlets were concentrated in white and higher-income census tracts, with fewer licensed outlets in areas with larger Latinx or Black populations, whereas unlicensed outlets were concentrated in areas with larger Latinx populations and areas with higher rates of poverty and unemployment. Similar to clustering of liquor stores in low-income neighborhoods (Laveist and Wallace 2000), we found that the majority of unlicensed outlets were in neighborhoods where the median household income was below $59,444. Despite countywide reductions in the number of unlicensed outlets, these illegal businesses remain clustered in Latinx, high poverty, and high unemployment areas where communities are disproportionately exposed to unregulated and high-risk cannabis products.

Beyond LA County, as noted earlier, Unger and colleagues showed that the presence of any unlicensed cannabis outlet in 2018 was more common in neighborhoods with larger Latinx populations in California (Unger et al. 2020). Additionally, they showed that neighborhoods across California with both licensed and unlicensed outlets had higher rates of poverty and larger Black and Asian populations. Yet, this statewide analysis did not detect any patterns between neighborhood demographic characteristics and areas with only licensed cannabis outlets in 2018, during the first year that licensed outlets were operating in California (Unger et al. 2020). It may be that the small number of licensed cannabis outlets in 2018 made it difficult to detect neighborhood patterns.

Our study showed that the number of licensed stores increased by 20% in just 1 year from 2019 to 2020. As the market stabilizes, monitoring the locations of both licensed and unlicensed outlets over time remains an important step in identifying neighborhoods that are disproportionately exposed to both licensed and unlicensed outlets, which can pose risks for population health. For example, cannabis storefront advertisement on licensed outlets may alter social norms and increase intentions to use cannabis among youth, and unregulated cannabis products sold by unlicensed outlets may cause unintentional injury to those who use these products (e.g., 2019 outbreak of lung damage (EVALI) was associated with unregulated cannabis vape cartridges sold at unlicensed outlets in Los Angeles) (The Associated Press 2020). In Los Angeles, living near licensed cannabis outlets was associated with heavy cannabis use and living near unlicensed outlets was also linked to heavy cannabis use, and symptoms of cannabis use disorder (Pedersen et al. 2021). In addition, storefront signage on medical dispensaries in Los Angeles—operating prior to licensing of cannabis outlets—was also associated with greater frequency of cannabis use among predominately underaged young adults (Shih et al. 2019). In Oregon, where licensed cannabis outlets have operated since 2015, outlet density was associated with increases in underage use over time among 6th, 8th, and 11th grade students (Paschall and Grube 2020).

These neighborhood-level analyses go beyond previous work by tracking changes in both licensed and unlicensed outlet locations over time, and results can inform discussions on how cannabis social equity programs at the local-level may affect potential applicants and their respective communities, but more information is still needed. Social equity programs have continued to struggle to award equity applicants with licenses that result in open businesses. Such impediments as the LA requirement to secure property prior to applying for a license is a direct contradiction of their goal to create an equitable legal cannabis environment (Gerber 2022). In San Francisco, the first equity applicant outlet was opened two years after the city began reviewing applications (Lekhtman 2020). Currently, 133 equity applications are waiting to be reviewed (City Performance Team 2019), and race/ethnicity data of licensees and applicants in San Francisco is unknown. What is clear is the lack of racial diversity among owners within the licensed cannabis industry. Drawing from other states with social equity provisions, 90% of cannabis licensees (e.g., owners, executives) in Massachusetts were white (Doonan and Johnson 2020)—compared to 71% of the census population (United States Census n.d.)—despite statewide efforts that prioritized applicants who have been disproportionately harmed by cannabis prohibition. Further, in Detroit, Michigan, over 80% of the population is Black yet only a couple cannabis business licenses were awarded to Black recipients (Gray 2019).

There are no publicly available data on characteristics of unlicensed outlet owners. We used neighborhood characteristics to infer populations most likely to live near, work at, or are customers of licensed and unlicensed outlets. Data on cannabis outlet owners and employees as well as those who applied for and did not receive a retail license could provide additional insights.

Using enforcement to shut down unlicensed outlets is one approach to move consumers to outlets selling regulated products, but this could exacerbate criminal justice and health inequities, especially if unlicensed sellers are low income or BIPOC. Employees of unlicensed outlets are arrested, as Amanda Lewis wrote for *Politico*, “Many of the illegal shops are in Black and Latino neighborhoods, with their employees vulnerable to arrest while owners are shielded behind shell companies. So as police and prosecutors attempt to crack down on unlicensed dispensaries, they appear to be reproducing the very social inequalities that legalization was supposed to fix” (Lewis 2021). Instead of using civil penalties or alternative enforcement measures (e.g., utility disconnection, padlocking or barricading properties (Title 2021)), another option is for jurisdictions to formally work with unlicensed outlets, to help them get licensed and provide amnesty during the transition. The city of San Francisco has used this amnesty model where preexisting medical dispensaries and cannabis outlets operating in a zoning-compliant location (e.g., 600 feet from schools or another outlet) were granted temporary permits from the city (City Performance Team 2019). Reducing barriers for unlicensed outlets to gain licenses could increase racial/ethnic diversity in the cannabis market, build economic wealth in marginalized communities, and reduce potentially negative population health impacts related to consumption of unregulated products. However, the overall impacts of social equity efforts will likely vary by jurisdiction and could be heavily influenced by federal changes in cannabis laws (Kilmer and Neel 2020). Going forward, comprehensive evaluations of social equity programs—from individual sellers to the communities living near outlets—are critical for understanding the impacts on low income and BIPOC populations.

## Data Availability

The datasets used and/or analyzed during the current study are available from the corresponding author on reasonable request.
